# Synergistic Interaction of CPP2 Coupled with Thiazole Derivates Combined with Clotrimazole and Antineoplastic Drugs in Prostate and Colon Cancer Cell Lines

**DOI:** 10.3390/ijms222111984

**Published:** 2021-11-05

**Authors:** Diana Duarte, Nuno Vale

**Affiliations:** 1 OncoPharma Research Group, Center for Health Technology and Services Research (CINTESIS), Rua Doutor Plácido da Costa, 4200-450 Porto, Portugal; up201201433@ff.up.pt; 2 Faculty of Pharmacy, University of Porto, Rua Jorge Viterbo Ferreira, 228, 4050-313 Porto, Portugal; 3 Department of Community Medicine, Health Information and Decision (MEDCIDS), Faculty of Medicine, University of Porto, Alameda Professor Hernâni Monteiro, 4200-319 Porto, Portugal

**Keywords:** drug combination, cell-penetrating peptides, thiazole derivates, clotrimazole, prostate cancer, colon cancer

## Abstract

Cell-penetrating peptides (CPPs) are small peptide sequences used mainly as cellular delivery agents that are able to efficiently deliver cargo into cells. Some CPPs also demonstrate intrinsic anticancer properties. Previously, our group developed a new family of CPP2-thiazole conjugates that have been shown to effectively reduce the proliferation of different cancer cells. This work aimed to combine these CPP2-thiazole conjugates with paclitaxel (PTX) and 5-fluorouracil (5-FU) in PC-3 prostate and HT-29 colon cancer cells, respectively, to evaluate the cytotoxic effects of these combinations. We also combined these CPP2-thiazole conjugates with clotrimazole (CLZ), an antifungal agent that has been shown to decrease cancer cell proliferation. Cell viability was evaluated using MTT and SRB assays. Drug interaction was quantified using the Chou–Talalay method. We determined that CPP2 did not have significant activity in these cells and demonstrate that N-terminal modification of this peptide enhanced its anticancer activity in both cell lines. Our results also showed an uneven response between cell lines to the proposed combinations. PC-3 cells were more responsive to the combination of CPP2-thiazole conjugates with CLZ than PTX and were more sensitive to these combinations than HT-29 cells. In addition, the interaction of drugs resulted in more synergism in PC-3 cells. These results suggest that N-terminal modification of CPP2 results in the enhanced anticancer activity of the peptide and demonstrates the potential of CPPs as adjuvants in cancer therapy. These results also validate that CLZ has significant anticancer activity both alone and in combination and support the strategy of drug repurposing coupled to drug combination for prostate cancer therapy.

## 1. Introduction

Cell-penetrating peptides (CPPs) are one of the most promising vehicles for the delivery of various types of cargo and have been extensively studied for the transport of different substrates to cancer cells [1]. CPPs are small peptides, usually 5–30 amino acids in length, and have the advantage of being able to translocate through the cell membrane via a non-toxic and apparently independent process of receivers or energy consumption [2]. CPPs are capable of transporting into the interior of living cells a wide variety of substrates such as drugs, peptides, proteins, liposomes, nanoparticles or even nucleic acids [3]. There is currently a large panoply of CPPs that can be distinguished as to their origin as peptides derived from proteins, chimeric peptides (resulting from the fusion of two natural sequences) or synthetic peptides (designed based on experimental and/or computational studies) [4].

CPPs can be classified into three classes: cationic, amphipathic and hydrophobic [5]. Currently, more than 100 different CPPs are patented with approximately 83% being cationic [6]. Hydrophobic CPPs are still very scarce and represent approximately 15% of all discoveries to date [7]. Although these peptides are rapidly assimilated, the different classes exhibit distinct behaviours, especially concerning endocytic internalisation.

Cationic CPPs are composed of positively charged residues such as arginine and lysine. Thus, the total charge of the peptide is always positive in physiological conditions. These basic residues are responsible for the first interaction of electrostatic nature with the plasma membrane, particularly with the negatively charged polysaccharides and lipids which eventually lead to the internalisation of the peptide in the cell [1]. Most cationic peptides are naturally occurring [6]; however, some artificial cationic peptides, such as homopolymers of lysine [8] and arginine [9], have already been synthesised. The use of cationic CPPs may be associated with the induction of side effects related to plasma membrane integrity and consequent cell viability, i.e., it may be associated with some degree of cell lysis [10].

One of the main applications of CPPs is in cancer therapy [3]. Since cancer is one of the greatest scourges in the world today, and since current therapy is strongly associated with many side effects [11], CPPs represent a very plausible alternative for the destruction of cancerous cells. To date, more than 1800 kinds of CPPs have been developed to deliver cargo from basic research to clinics in therapeutics delivery, gene editing and cell imaging [12].

Although CPPs are commonly used for the transport of different cargo, there are some specific amino acid sequences with intrinsic activity against tumour cells. For example, p28 (LSTAADMQGVVTDGMASGLDK-DYLKPDD) is a CPP that binds to wild-type and mutated p53, increasing its intracellular levels and inhibiting cancer cell proliferation. Indeed, this was observed in in vitro studies performed with colon, breast, ovarian and other solid tumour cells [13]. Supported by these findings, a phase I clinical trial (NCT00914914) was concluded in 2014, where p28 was tested against a range of p53+ solid tumours. It was found that the peptide was well tolerated, and no patients exhibited any dose-limiting toxicities (DLTs), significant adverse events or immune response (IgG). Additionally, there was evidence of antitumour activity indicating a highly favourable therapeutic index [14].

Another study attempted to describe artificial CPPs that were selectively and efficiently incorporated into human tumour cells according to their lineage [15]. The authors obtained ten representative tumour lineage homing cell-penetrating peptides by a screening of a random peptide library constructed using messenger RNA display technology. Among the results, they were able to target specific tumour cell types with different CPPs. One of those CPPs was FITC–CPP2 (a conjugated form of CPP2 with fluorescein isothiocyanate), which showed highly efficient permeation of the colon adenocarcinoma cell line, LoVo, without any significant uptake by the other tumour cell lines. This strong permeation was corroborated in three additional colon adenocarcinoma lines, Sw620, Colo320 and WiDr, whereas other lineages, such as MCF-7 and NHDF, did not show significant permeation [15]. Despite the promising results, no CPPs or CPP/cargo complexes have yet been approved by the US Food and Drug Administration (FDA) [3].

**Scheme 1:**
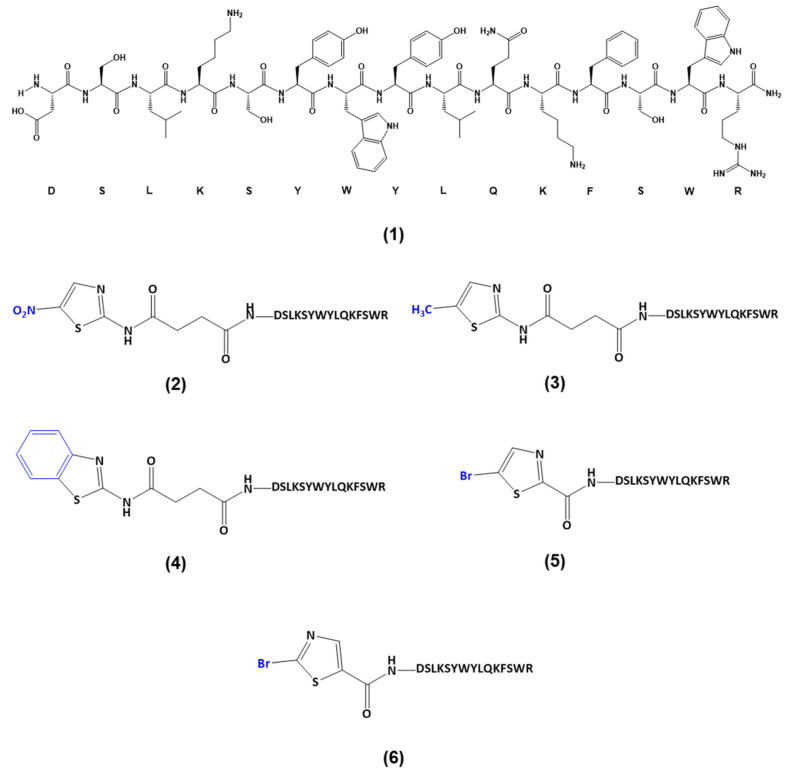
Structure of the CPP2–thiazole conjugates previously synthesised by our group [16] and used in this work: (**1**) CPP2; (**2**) NTZ-C2-CPP2; (**3**) MTZ-C2-CPP2; (**4**) BenzoTZ-C2-CPP2; (**5**) BTZCA-CPP2; (**6**) BTZ5CA-CPP2.

Based on these findings, our group previously reported the synthesis of novel CPP2–thiazole conjugates (Scheme 1) and evaluated their anticarcinogenic properties using Caco-2 colon and A549 lung cancer cells [16]. We used several thiazole conjugates: 2-amino-5-nitrothiazole (NTZ, **2**, Scheme 1), 2-amino-5-methylthiazole (MTZ, **3**, Scheme 1), 2-aminobenzothiazole (BenzoTZ, **4**, Scheme 1), 5-bromothiazole-2-carboxylic acid (BTZCA, **5**, Scheme 1) and 2-bromothiazole-5-carboxylic acid (BTZ5CA, **6**, Scheme 1). Of these, the first three were coupled to CPP2 by a linker (i.e., succinic anhydride); in contrast, BTZCA and BTZ5CA were directly coupled to CPP2. Our results have shown these conjugates have promising anticancer activity against these cancer cell lines [16].

Conventional chemotherapy has the main disadvantage of not being specific for tumour cells, acting both on normal and carcinogenic cells. In addition to its numerous side effects, the concentration of drug that reaches the tumour site is quite low. Thus, the strategy of combining anticancer agents with CPPs may be very beneficial, since this synergism may increase the permeability of membranes for therapeutic agents and direct them toward the tumour’s location, allowing for an increase in the concentration of drugs where they are strictly necessary, leading to an increase in treatment efficacy.

**Figure 1:**
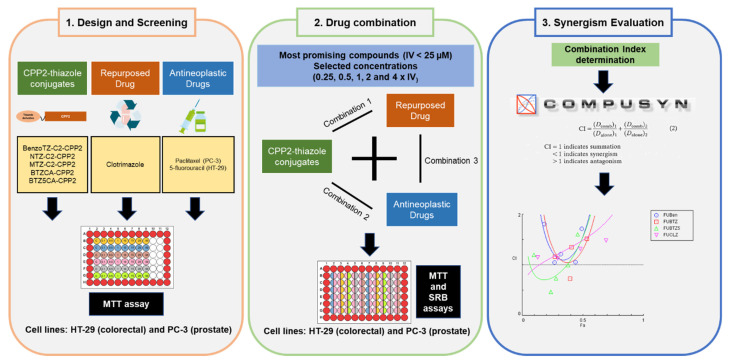
Representation of the study design.

In this work, we aimed to combine the previously synthesised CPP2–thiazole conjugates with paclitaxel (PTX) and 5-fluorouracil (5-FU) in PC-3 prostate and HT-29 colon cancer cells, respectively, to investigate the cytotoxic effects of these combinations for cancer treatment. PTX and 5-FU were chosen because they are antineoplastic drugs commonly used for prostate and colon cancer therapy. Based on our previous experience using repurposed drugs in combinatorial therapies [17,18], and in a recent study where it was found that PTX combined with clotrimazole (CLZ), an antifungal agent, acted synergistically against breast cancer cells by increasing oxidative stress, reducing glucose uptake and enhancing genotoxicity [19], we also evaluated the combination of CPP2–thiazole conjugates and the reference drugs with CLZ. The study design is summarised in Figure 1.

## 2. Results

### 2.1. Cytotoxic Effect of CPP2–Thiazole Conjugates, Reference Drugs (PTX or 5-FU) and CLZ on HT-29 and PC-3 Cells

**Figure 2:**
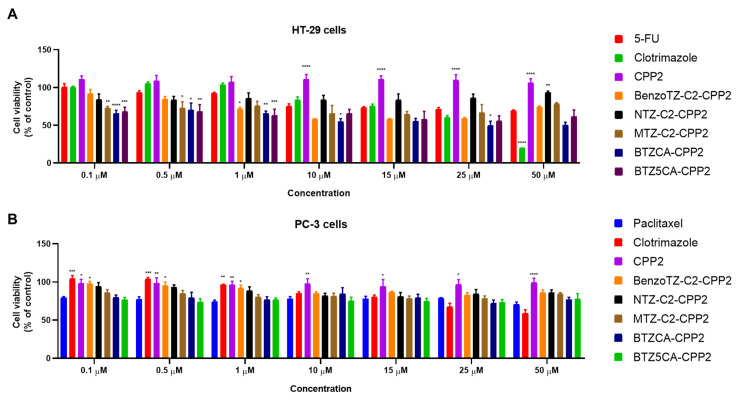
Cytotoxic effects of 5-FU or PTX, CLZ, CPP2 and CPP2–thiazole conjugates on the viability of HT-29 (**A**) and PC-3 (**B**) cells, respectively. Cell viability was assessed by MTT assay after 48 h of exposure to each treatment. The results are expressed in the percentage of cell viability relative to untreated control cells. *, **, *** and **** indicate *p* < 0.05, *p* < 0.01, *p* < 0.001 and *p* < 0.0001, respectively, when compared with 5-FU or PTX. All data are presented as the mean ± SEM of three independent experiments.

PTX and 5-FU are well-recognised anticancer compounds used in the chemotherapy of prostate and colon cancer, respectively. Clotrimazole has also been reported to have antitumor activity with minimal effect on normal cells. We first investigated the effect of each drug individually on HT-29 colon and PC-3 prostate cells using increasing concentrations (0.1–50 µM) of CPP2, five CPP2–thiazole conjugates and clotrimazole for 48 h. Cell viability was evaluated by MTT assay. HT-29 and PC-3 cells were also treated with 5-FU and PTX, respectively (Figure 2). The results regarding HT-29 cells demonstrate that BTZCA–CPP2 and BTZ5CA–CPP2 are the most promising CPP2 conjugates for decreasing the viability of these cells, with enhanced anticancer activity compared to 5-FU. Clotrimazole treatment in concentrations above 25 µM also demonstrated more cytotoxicity than 5-FU alone, with 50 µM treatment resulting in more viability reduction than the previous referred CPP2 conjugates. Treatment with CPP2 did not result in a significant reduction in the viability of HT-29 cells. Cells treated BenzoTZ-C2–CPP2, NTZ-C2–CPP2 and MTZ-C2–CPP2 did not result in significant changes compared to the treatment with 5-FU (Figure 2A). In PC-3 prostate cancer cells, CPP2 and CPP2–thiazole conjugates did not significantly reduce cell viability compared to the treatment with 5-FU. At higher concentrations (>25 µM), clotrimazole treatment resulted in an enhancement of the number of dead cells compared to PTX (Figure 2B).

**Figure 3:**
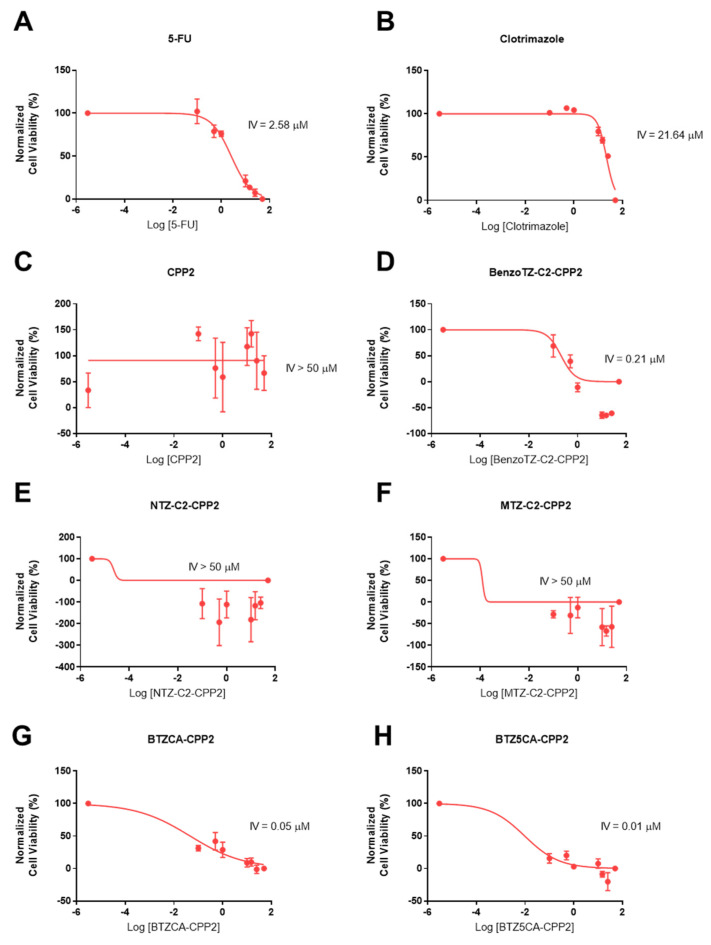
Concentration–response curves of 5-FU (**A**), CLZ (**B**), CPP2 (**C**), BenzoTZ-C2-CPP2 (**D**), NTZ-C2-CPP2 (**E**), MTZ-C2-CPP2 (**F**), BTZCA-CPP2 (**G**) and BTZ5CA-CPP2 (**H**) on HT-29 cells based on the results obtained by MTT assay. The results represent the mean ± SEM of three independent experiments performed in triplicate.

**Figure 4:**
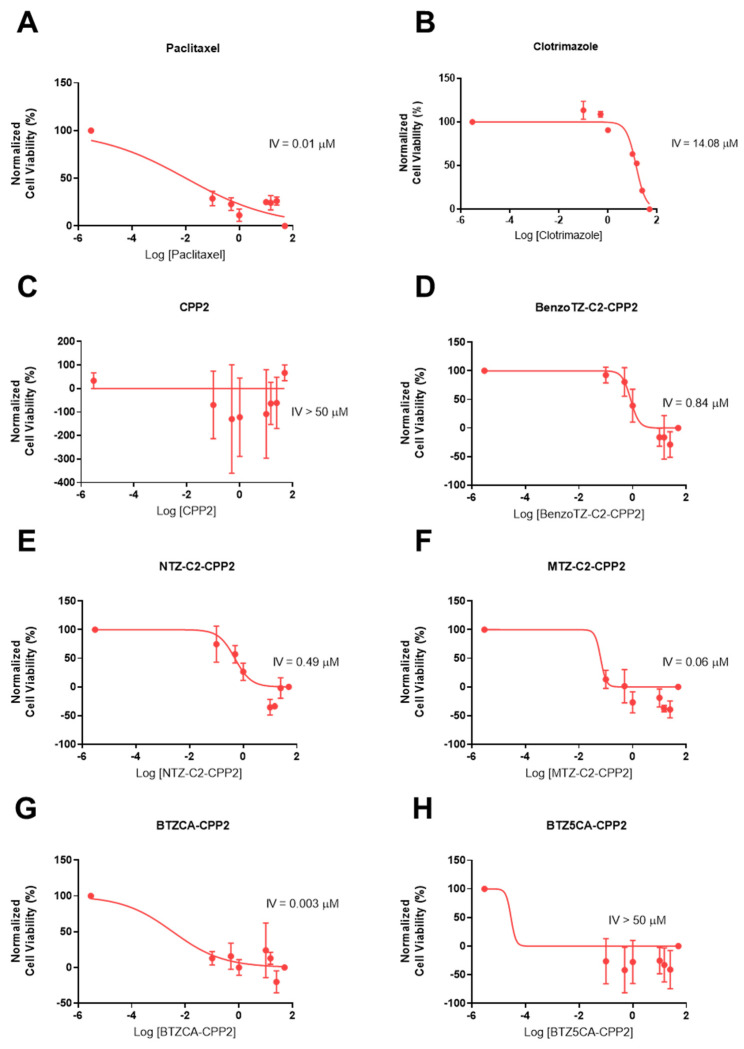
Concentration–response curves of PTX (**A**), CLZ (**B**), CPP2 (**C**), BenzoTZ-C2-CPP2 (**D**), NTZ-C2-CPP2 (**E**), MTZ-C2-CPP2 (**F**), BTZCA-CPP2 (**G**) and BTZ5CA-CPP2 (**H**) on PC-3 cells based on the results obtained by MTT assay. The results represent the mean ± SEM of three independent experiments performed in triplicate.

Based on the previous MTT results, we found that most peptides did not achieve a 50% cell inhibition in any line, so we next calculated an intermediate value (IV) for each compound individually by nonlinear regression using GraphPad software for both cell lines (Table 1, Figure 3 and Figure 4). This IV was obtained by plotting the logarithm of concentration against the normalised values of cell viability, as in a dose–response curve, but did not necessarily represent the IC_50_ of each compound, as these peptides had reached a plateau of inhibition. It is necessary for this IV to have a method of comparison for the combination of experiments. Clotrimazole treatment resulted in higher IV values in HT-29 than in PC-3 cells, demonstrating better anticancer effects in prostate cancer. 5-FU’s and PTX’s half inhibitory concentrations were 2.58 and 0.01 µM in HT-29 and PC-3 cells, respectively. For both cell lines, CPP2 treatment resulted in IV values higher than 50 µM, demonstrating a lack of activity in both cell lines. In HT-29 cells, treatment with NTZ– and MTZ-C2–CPP2 also resulted in a poor anticancer effect. BenzoTZ-C2–CPP2 and the two conjugates without a linker (i.e., BTZCA–CPP2 and BTZ5CA–CPP2) resulted in IV values lower than 1 µM in HT-29 cells. In PC-3 cells, the conjugates BenzoTZ–, MTZ– and NTZC2–CPP2 and BTZCA–CPP2 also resulted in low IV values. CPP2 conjugates with an IV lower than 20 µM were selected for combination with clotrimazole and antineoplastic drugs.

### 2.2. Cytotoxic Effect of Selected Combinations of CPP2–Thiazole Conjugates plus Reference Drugs (PTX or 5-FU) on HT-29 and PC-3 Cells

In vitro cytotoxic effects of the selected CPP2–thiazole conjugates combined with 5-FU or PTX were assessed by MTT and SRB assays in two different cell lines, HT-29 and PC-3, respectively. For that, five concentrations were selected, around the IV (0.25, 0.5, 1, 2, and 4 times IV) of each drug, both alone and combined, according to our previous combination model [18].

**Figure 5:**
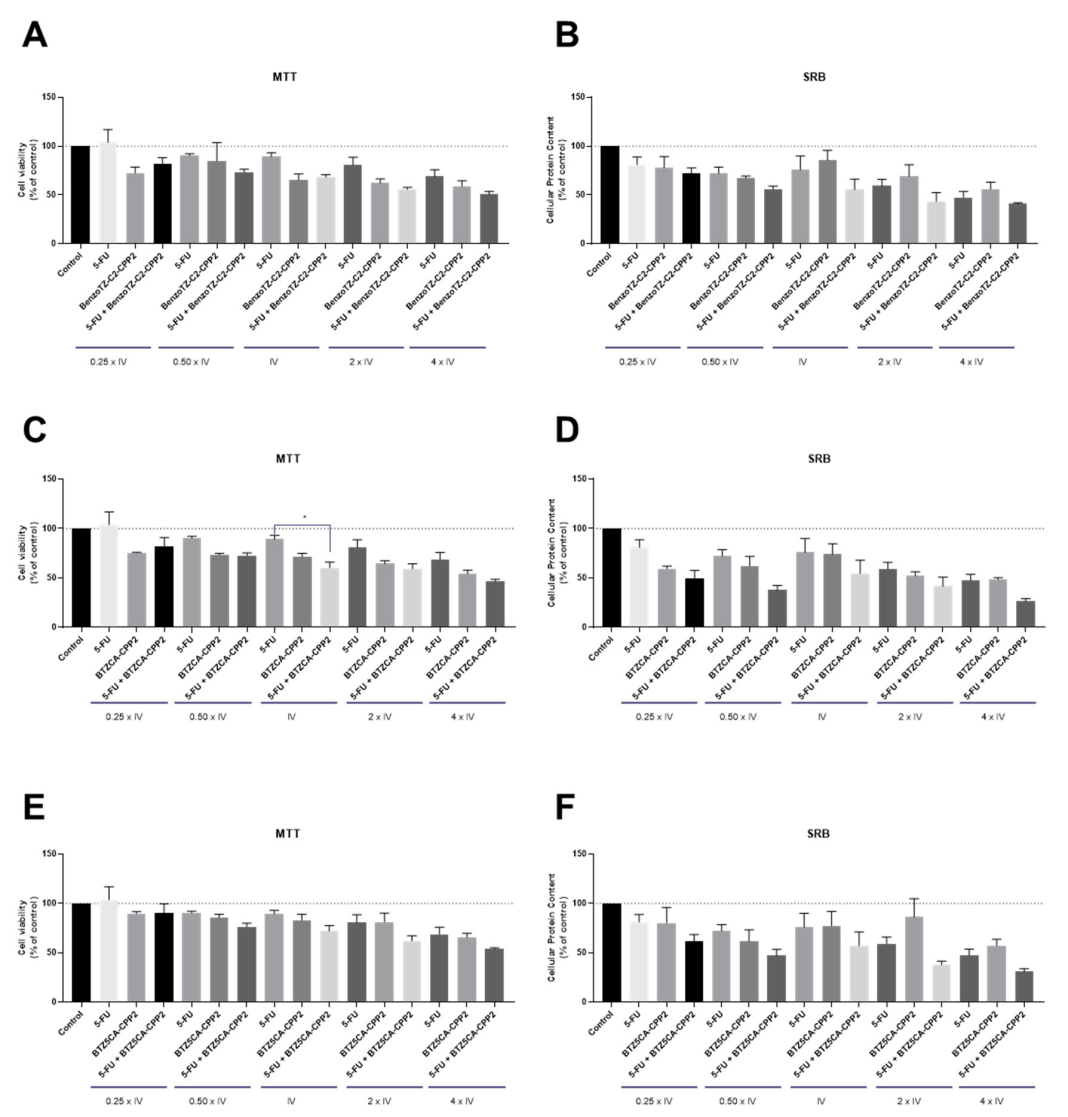
Cytotoxic effects of the combination of 5-FU with (**A**,**B**) BenzoTZ-C2-CPP2, (**C**,**D**) BTZCA-CPP2 and (**E**,**F**) BTZ5CA-CPP2 on the viability (**right** panel) and cell protein (**left** panel) content of HT-29 cells. Cell viability and protein content were assessed by MTT and SRB assays, respectively, after 48 h of exposure to each treatment. Results are expressed in the percentage of cell viability relative to untreated control cells. * Indicates *p* < 0.05 when compared to control. All data are presented as the mean ± SEM of three independent experiments.

For the HT-29 cell line (Figure 5) and according to Table 1, three different CPP2–thiazole conjugates were selected for combination with 5-FU: BenzoTZ-C2–CPP2, BTZCA–CPP2 and BTZ5CA–CPP2. 5-FU was chosen because this drug is commonly used for colorectal cancer therapy. The results between the MTT and SRB assays were in agreement and demonstrated a lack of significant antitumor activity for BenzoTZ-C2–CPP2 and BTZ5CA–CPP2 both alone and combined with 5-FU in HT-29 colon cancer cells. Moreover, treatment with 5-FU alone did not show significant modulation of the viability of these cells. Only cells treated with 5-FU+BTZCA–CPP2 demonstrated a significant reduction in cell viability, compared to 5-FU in cells exposed to 5-FU and BTZCA–CPP2 in the concentration of the IV of each drug, by MTT assay. Overall, these results suggest that combination with 5-FU is not advantageous over treatment with CPP2–thiazole derivatives alone.

**Figure 6:**
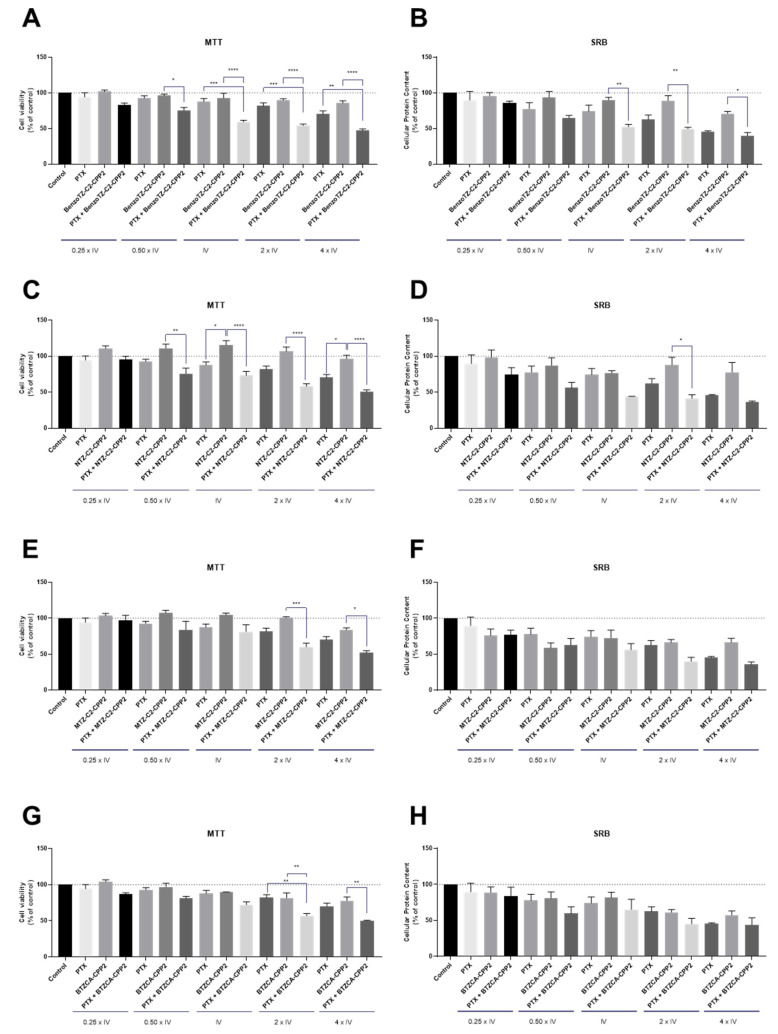
Cytotoxic effects of the combination of PTX with (**A**,**B**) BenzoTZ-C2-CPP2, (**C**,**D**) NTZ-C2-CPP2, (**E**,**F**) MTZ-C2-CPP2 and (**G**,**H**) BTZCA-CPP2 on the viability (**right** panel) and cell protein (**left** panel) content of PC-3 cells. Cell viability and protein content were assessed by MTT and SRB assays, respectively, after 48 h of exposure to each treatment. Results are expressed in the percentage of cell viability relative to untreated control cells. *, **, *** and **** indicate *p* < 0.05, *p* < 0.01, *p* < 0.001 and *p* < 0.0001, respectively, when compared to the control. All data are presented as the mean ± SEM of three independent experiments.

Regarding the PC-3 cell line, treatment was conducted with the combination of the previously selected CPP2–thiazole conjugates (i.e., BenzoTZ-C2–CPP2, NTZ-C2–CPP2, MTZ-C2–CPP2 and BTZCA–CPP2) with PTX, an antineoplastic drug used for prostate cancer therapy. Cells were exposed to each drug, both alone and in combination, based on our previous combination model [18] in line with the experiments described above for HT-29 cells. Cell viability was evaluated by MTT assay, and cellular protein content was assessed by SRB assay with exposure for 48 h (Figure 6). The results for the PC-3 cells suggest that both cell-based assays were in agreement for all treatments. Compared to the results presented in Figure 5, we found that PTX enhanced anticancer activity in PC-3 more than 5-FU in HT-29 cells. Treatment with each CPP2–thiazole derivative in this cell line did not result in significant anticancer activity. These results also demonstrate enhanced results for the pairs of drugs combined in prostate cancer cells than in HT-29 cells, with BenzoTZ-C2–CPP2 + PTX resulting in a significant reduction in cell viability, compared to PTX and BenzoTZ-C2–CPP2 alone, for the concentrations of IV and higher. The combination of reference drug with NTZ-C2–CPP2 and MTZ-C2–CPP2 only resulted in significant cell death compared to each CPP2–thiazole derivative alone. The combination of BTZCA–CPP2 + PTX in concentrations two times that of the IV also decreased cell viability to a percentage that statistically differed from each drug alone.

### 2.3. Cytotoxic Effect of Selected Combinations of CPP2–Thiazole Conjugates plus Repurposed Drug (CLZ) on HT-29 and PC-3 Cells

Regarding CPP2–thiazole conjugates combined with reference drugs, it did not result in many significant results, mainly in HT-29 cells. We proposed another combination model using a repurposed drug (CLZ). CLZ is an antifungal drug widely used to treat common fungal infections. Several studies have demonstrated that this drug also possesses anticancer activity. A combination model was also created based on our previous works and similar to that described above. We also evaluated cell viability and cellular protein content by MTT and SRB assays for a treatment period of 48 h.

**Figure 7:**
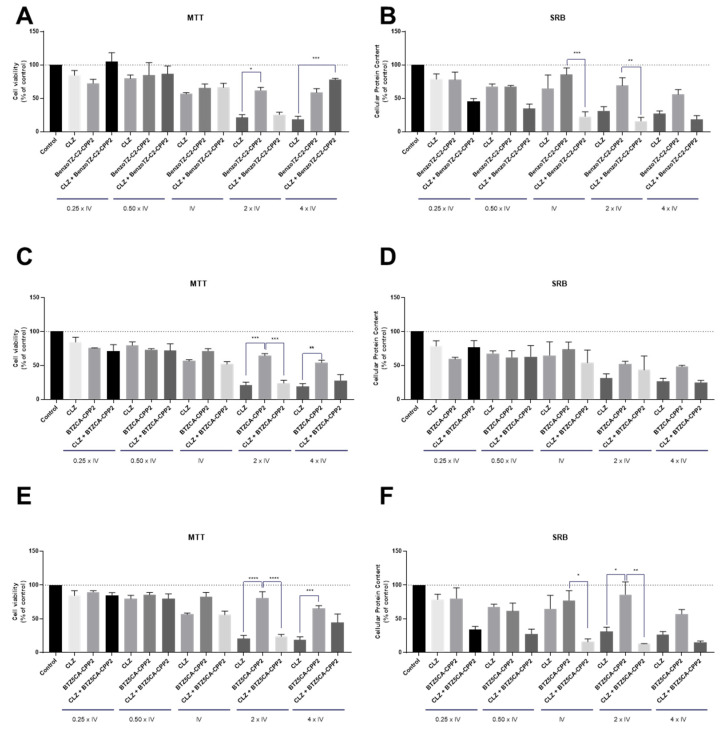
Cytotoxic effects of the combination of CLZ with (**A**,**B**) BenzoTZ-C2-CPP2, (**C**,**D**) BTZCA-CPP2 and (**E**,**F**) BTZ5CA-CPP2 on the viability (**right** panel) and cell protein (**left** panel) content of HT-29 cells. Cell viability and protein content were assessed by MTT and SRB assays, respectively, after 48 h of exposure to each treatment. Results are expressed in the percentage of cell viability relative to untreated control cells. *, **, *** and **** indicate *p* < 0.05, *p* < 0.01, *p* < 0.001 and *p* < 0.0001, respectively, when compared to the control. All data are presented as the mean ± SEM of three independent experiments.

Regarding HT-29 cells, the results suggest that CLZ has efficacy in the reduction of viability and protein synthesis of these cells, mainly in concentrations of IV and higher, demonstrating a greater anticancer potential than 5-FU in these cells (Figure 7). Nonetheless, the combination of CPP2–thiazole derivates has not demonstrated promising results compared to CLZ alone. Indeed, our results suggest that the anticancer activity observed in the combinations resulted from the activity of CLZ and not from the thiazole derivate.

**Figure 8:**
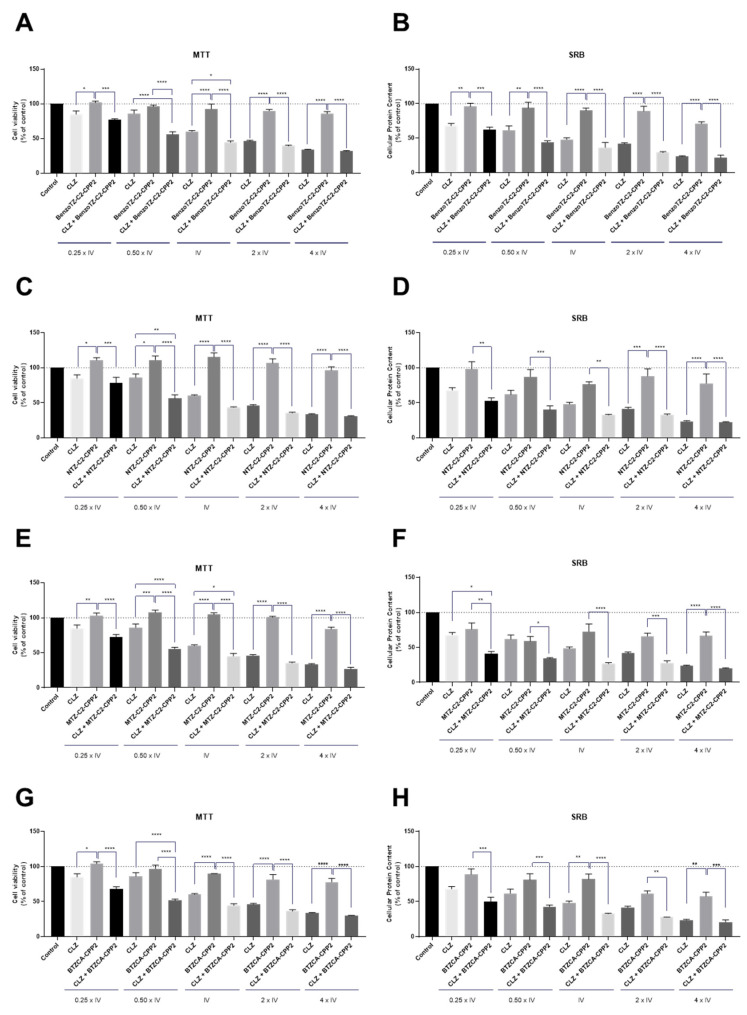
Cytotoxic effects of the combination of CLZ with (**A**,**B**) BenzoTZ-C2-CPP2, (**C**,**D**) NTZ-C2-CPP2, (**E**,**F**) MTZ-C2-CPP2 and (**G**,**H**) BTZCA-CPP2 on the viability (**right** panel) and cell protein (**left** panel) content of PC-3 cells. Cell viability and protein content were assessed by MTT and SRB assays, respectively, after 48 h of exposure to each treatment. Results are expressed in the percentage of cell viability relative to the untreated control cells. *, **, *** and **** indicate *p* < 0.05, *p* < 0.01, *p* < 0.001 and *p* < 0.0001, respectively, when compared to the control. All data are presented as the mean ± SEM of three independent experiments.

Concerning PC-3 cells, our results demonstrated promising drug pairs for cancer therapy (Figure 8). Both the MTT and SRB results were similar and showed that CLZ had indeed great potential for drug repurposing for prostate cancer therapy. We also found that CPP2–thiazole derivatives alone did not demonstrate great results in the reduction of viable cells for the selected range of concentrations. CLZ combined with BenzoTZ-C2–CPP2 is advantageous over each drug alone, even in concentrations above the IV, promoting cell death in these cells. Combination with both NTZ–, MTZ-C2–CPP2 and BTZCA–CPP2 also resulted in a significant reduction in the cell viability in concentrations of 0.50 times the IV by MTT assay, with a statistically significant reduction in viable cells compared to CLZ and each thiazole derivative alone.

### 2.4. Cytotoxic Effect of Reference Drugs (PTX or 5-FU) plus the Repurposed Drug (CLZ) on HT-29 and PC-3 Cells

**Figure 9:**
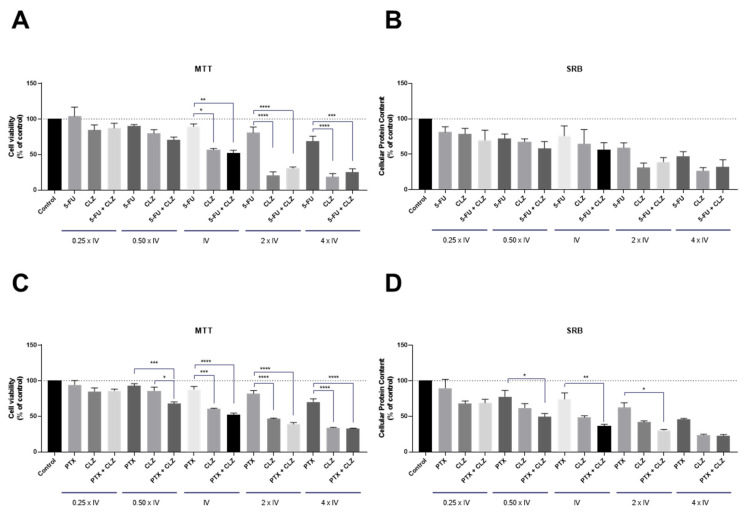
Cytotoxic effects of the combination of CLZ with (**A**,**B**) 5-FU and (**C**,**D**) PTX on the viability (**right** panel) and cell protein (**left** panel) content of HT-29 and PC-3 cells, respectively. Cell viability and protein content were assessed by MTT and SRB assays, respectively, after 48 h of exposure to each treatment. The results are expressed in the percentage of cell viability relative to untreated control cells. *, **, *** and **** indicate *p* < 0.05, *p* < 0.01, *p* < 0.001 and *p* < 0.0001, respectively, when compared to the control. All data are presented as the mean ± SEM of three independent experiments.

Moreover, we also evaluated the combination of CLZ with PTX or 5-FU in PC-3 and HT-29 cells, respectively, to evaluate if this combination can also be promising for cancer therapy. Interestingly, results in both cell lines were very similar and demonstrated that CLZ enhanced the anticancer efficacy more than PTX and 5-FU (Figure 9). In addition, the results regarding the combination of the repurposed drug with each reference drug seemed to suggest that the anticancer activity from the combination was a result of the activity of CLZ alone, not demonstrating the benefits for its use in oncotherapy.

### 2.5. Morphological Analysis of HT-29 and PC-3 Cells Treated with CPP2–Thiazole Derivatives Both Alone and Combined

**Figure 10:**
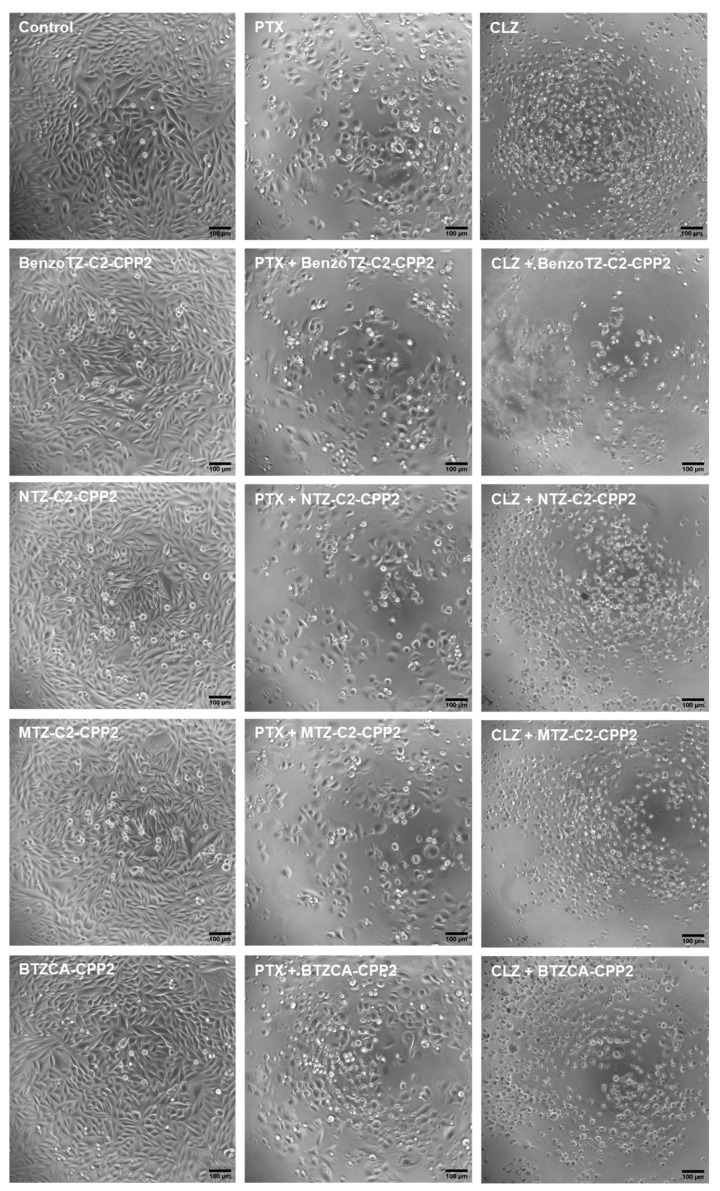
Microscopic visualisation of PC-3 treated with CPP2-thiazole derivates, both alone and combined with PTX and CLZ, at concentrations of 4 × IV. Representative images were obtained with a high contrast brightfield objective (10×) (LionHeart FX Automated Microscope) from three independent experiments. Scale bar: 100 µM.

All previous treatments were confirmed by morphological analysis, both in PC-3 and HT-29 cells. Regarding PC-3 cells (Figure 10), compared to the control cells, differences both in the number of cells and morphology were visible in several treatments, in agreement with the previous results. PTX treatment resulted in a decrease in the number of cells per well, compared to the control. The CLZ treatment induced a more aggressive phenotype in these cells, with most of them being smaller and rounder, indicative of cell death. All treatments with CPP2–thiazole conjugates alone did not result in significant changes in cell morphology compared to the control cells, reinforcing the previous results obtained by MTT and SRB. For all combination treatments, CPP2–thiazole conjugates plus CLZ resulted in more pronounced changes in cell number and morphology than with PTX.

**Figure 11:**
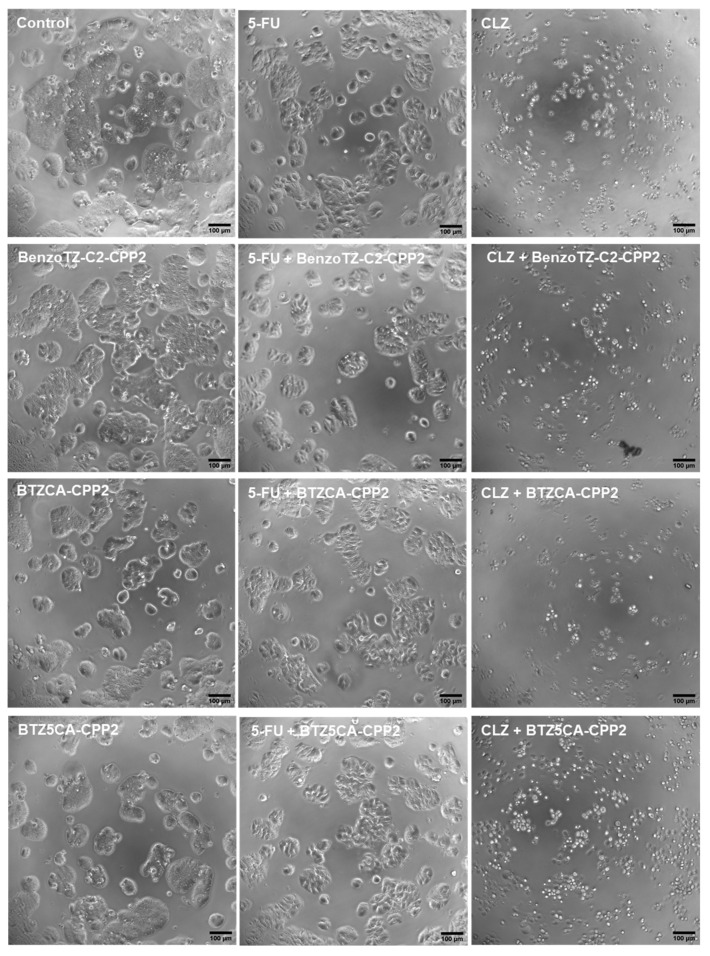
Microscopic visualisation of HT-29 treated with CPP2-thiazole derivates, both alone and combined with 5-FU and CLZ, at concentrations of 4 × IV. Representative images were obtained with a high contrast brightfield objective (10×) (LionHeart FX Automated Microscope) from three independent experiments. Scale bar: 100 µM.

Analysing the HT-29 cells (Figure 11), the results followed the same tendency as the previous results for the PC-3 cells. CLZ shows more potency than 5-FU in reducing cell numbers and promoting cell death. No significant differences were noted among HT-29 cells treated with the CPP2–thiazole derivates alone compared to the control cells. Combination with 5-FU decreased cell number per well, but cell morphology was still maintained. Combination with CLZ was very strong in reducing the number of viable cells and resulted in changes in the number and phenotype of these cells. Altogether, these results support the previous results obtained in the MTT and SRB assays.

### 2.6. Evaluation of the Drug Interaction of Reference Drugs (PTX or 5-FU) and CLZ plus CPP2–Thiazole Derivatives on HT-29 and PC-3 Cells

Based on the MTT results, we next evaluated the interaction between the drugs applied in combination to both cell lines to confirm if there were synergic pairs in the previous combinations. The combination index (CI) was calculated using the Chou–Talalay method. CI was plotted on the *y*-axis as a function of effect level (Fa) on the *x*-axis. CI < 1, =1 or >1 means synergism, additivity or antagonism, respectively. The fractional effect (Fa) is a parameter between 0 and 1, where 0 means the combination did not affect cell viability, and 1 means the combination produced a full effect on decreasing cell viability.

**Figure 12:**
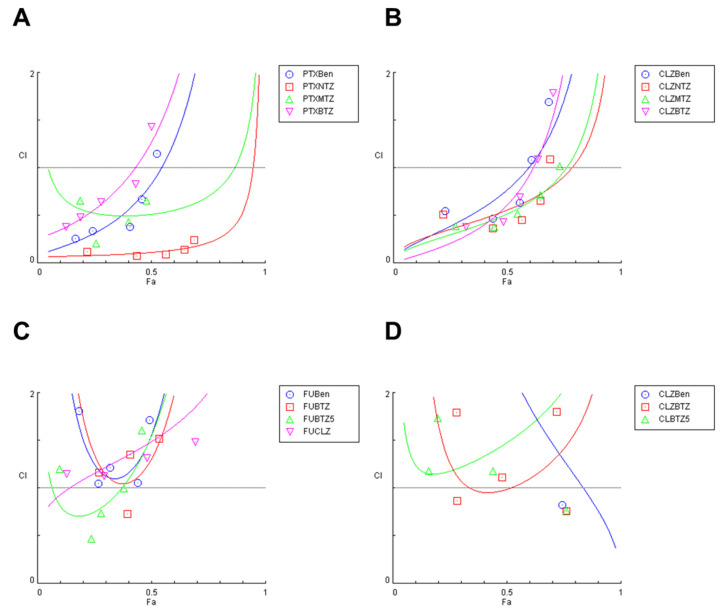
Chou–Talalay method Fa–CI plot of (**A**) PTX and (**B**) CLZ combined with CPP2–thiazole conjugates in PC-3 cells and of (**C**) 5-FU and (**D**) CLZ combined with CPP2–thiazole conjugates in HT-29 cells. CI is plotted on the *y*-axis as a function of effect level (Fa) on the *x*-axis to assess drug synergism. CI < 1, CI = 1 and CI > 1 indicate synergism, additivity, and antagonism, respectively. PTXBen: PTX + BenzoTZ-C2-CPP2; PTXNTZ: PTX + NTZ-C2-CPP2; PTXMTZ: PTX + MTZ-C2-CPP2; PTXBTZ: PTX + BTZCA-CPP2; CLZBen: CLZ + BenzoTZ-C2-CPP2; CLZNTZ: CLZ + NTZ-C2-CPP2; CLZMTZ: CLZ + MTZ-C2-CPP2; CLZBTZ: CLZ + BTZCA-CPP2; FUBen: 5-FU + BenzoTZ-C2-CPP2; FUBTZ: 5-FU + BTZCA-CPP2; FUBTZ5: 5-FU + BTZ5CA-CPP2; FUCLZ: 5-FU + CLZ; CLZBen: CLZ + BenzoTZ-C2-CPP2; CLZBTZ: CLZ + BTZCA-CPP2; CLZBTZ5: CLZ + BTZ5CA-CPP2.

**Figure 13:**
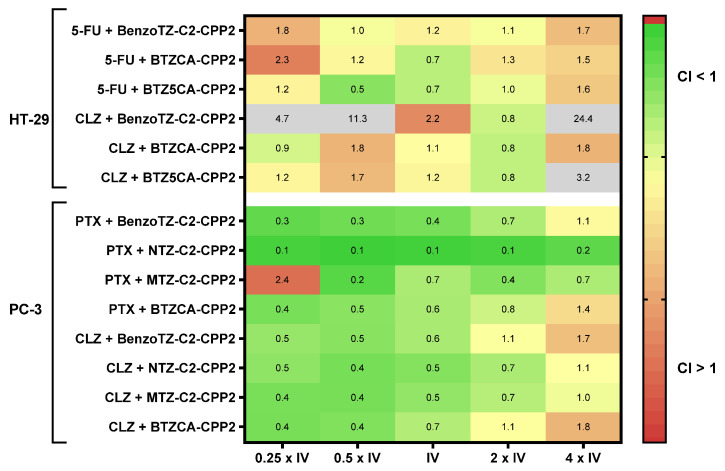
Synergy heat map compiling the CI values in both cell lines treated with the combination of CPP2–thiazole derivates plus the reference drug or CLZ. The squares in the figure indicate antagonism (CI > 1.2, red), additivity (0.9 < CI < 1.1, yellow) and synergism (CI < 0.9, green).

The combination of PTX + CPP2–thiazole derivatives in PC-3 cells demonstrated more synergism than the combination of 5-FU + CPP2–thiazole derivatives in HT-29 cells (Figure 12). In addition, the combination of CLZ with CPP2–thiazole derivatives in PC-3 cells resulted in more CI values under one than the same combinations in HT-29 cells. Comparing the combination of CLZ and the reference drug with CPP2–thiazole conjugates in PC-3 cells, these results demonstrate more synergistic pairs in combination with PTX. Regarding PC-3 cells, and although the MTT results were more promising in combination with CLZ, the following results are easily explained: as synergy represents a potentiation of the effect compared to each drug alone, and as PTX demonstrated worse anticancer activity than CLZ alone in these cells, it is understandable that combination with PTX represents more synergism than with CLZ, as the potentiation in the combination compared to each drug alone was lower with the last drug. Altogether, these results demonstrate that PC-3 treated with these combinations resulted in more synergistic pairs than HT-29, with PTX + NTZ-C2-CPP2 being the only pair with a synergic effect for all concentrations tested (Figure 13).

## 3. Discussion

CPPs are small peptide sequences that have been studied mainly for the transport of different cargo from the extra- to intracellular environment without damaging the membrane of the cells. Most CPPs are composed of positively charged amino acids that interact with the negatively charged phospholipids of the cell membrane, interfering temporarily with their stability and enhancing their permeability. CPPs have been studied for different medical fields such as cancer and antibiotic infections.

Several efforts have been made to improve some characteristics of CPPs. N-terminal modifications are one of the most popular strategies that can help to enhance the peptide ability for cargo transport and/or increasing the intrinsic activity of these peptides. In a previous study, FITC–CPP2 (a conjugated form of CPP2 with fluorescein isothiocyanate) showed highly efficient permeation of the colon adenocarcinoma cell line, LoVo, without any significant uptake by the other tumour cell lines. This strong permeation was corroborated in three additional colon adenocarcinoma lines: Sw620, Colo320 and WiDr. Following this study, our group synthesised CPP2 and performed N-terminal modifications using different thiazole derivatives, as there was evidence that this moiety displayed anticancer effects. We successfully synthesised six different CPP2–thiazole conjugates and tested them against Caco-2 colon and A549 lung cell lines and found these conjugates were promising for cancer therapy.

In this work, we aimed to evaluate the combination of these previously synthesised CPP2–thiazole conjugates with reference drugs in two different cell lines: HT-29 colon and PC-3 prostate cancer. 5-FU was chosen as a reference drug for HT-29 cells and PTX for PC-3 cells. These conjugates were also combined with CLZ, a widely used antifungal drug, to evaluate if the repurposing of this drug could be useful for cancer therapy. In a recent study, it was found that PTX combined with CLZ acts synergistically against breast cancer cells by increasing oxidative stress, reducing glucose uptake and enhancing genotoxicity.

The drug repurposing represents a faster and cheaper strategy for identifying new potential anticancer therapies. As repurposed drugs have previously been approved for other diseases and have well-established clinical profiles, their approval for another indication is easier than the development of novel drugs. Moreover, the strategy of drug combination allows for the reduction in drug concentrations needed for therapeutical effects.

CPP2–thiazole conjugates, each reference drug and CLZ were first screened by MTT assay in these two cell lines in a wide range of concentrations (1–50 µM) to determine their half-maximum concentration. Our results have demonstrated that CPP2 lacks anticancer activity against both cell lines. BenzoTZ-C2–CPP2 and BTZCA–CPP2 display promising antitumor activity in both cell lines, demonstrating that N-terminal modification of CPP2 resulted in the enhanced intrinsic anticancer activity of this peptide. CLZ treatment resulted in a strong reduction in the number of viable cells in both cell lines, with better results than both PTX and 5-FU. This demonstrates that the repurposing of CLZ is plausible and that this drug can represent a promising candidate for cancer therapy. The most promising compounds (IV < 20 µM) were selected for combination with the reference drug and CLZ, following our combination model previously described. Cell viability and cell protein content was evaluated by MTT and SRB assays, respectively. We found that the combination of PTX with CPP2–thiazole conjugates in PC-3 cells resulted in a stronger reduction in viable cells than with 5-FU in HT-29 cells, compared to both compounds alone. We also found that CLZ combined with these conjugates induced a more pronounced reduction in cell viability in PC-3 than HT-29 cells. Cell morphology was also analysed after each treatment, and it was confirmed visually that these results were in agreement with SRB and MTT assays. Next, synergism was evaluated using the Chou–Talalay method which is based on the median-effect equation, derived from the mass-action law principle. This unified theory encompasses the Michaelis–Menten, Hill, Henderson–Hasselbalch and Scatchard equations in biochemistry and biophysics and provides a quantitative definition for an additive effect (CI = 1), synergism (CI < 1) and antagonism (CI > 1) in drug combinations [20]. In line with the previous results, we found more synergic pairs in the PC-3 cell line.

This work evaluated, for the first time, the combination of CPPs with reference and repurposed drugs. We demonstrated that these CPP2–thiazole conjugates can be successfully combined with both PTX and CLZ, resulting in enhanced anticancer effects compared to each drug alone and synergistically inhibit cancer cell proliferation, mainly in PC-3 cells. Using peptides with penetrating properties, the interaction with drugs can be enhanced and thus justify the significant synergistic effect observed. Also, as different cells lines are metabolically different and have specific characteristics, more research should be conducted on other cell lines such as breast and lung. Deeper mechanistic studies should also be performed to evaluate the anticancer mechanisms underlying these drugs and these combinations. These are preclinical results that should be further confirmed in animal models and clinical trials. Our results demonstrate that the combination of CPP2–thiazole conjugates may lead to novel therapeutic strategies for prostate cancer therapy.

## 4. Materials and Methods

### 4.1. CPP2 and CPP2–Thiazole Conjugate Synthesis

#### 4.1.1. Reagents and Solvents

All solvents used in this study were of analytical grade. Reagents and solvents were purchased from Novabiochem (Fmoc-amino acids and Fmoc-Rink Amide MBHA resin), Merck (TFA and other solvents), Sigma–Aldrich (coupling agents, piperidine, *N*-ethyl-*N*,*N*-diisopropylamine (DIEA) and all thiazole derivatives).

#### 4.1.2. Synthesis of CPP2

The first peptide to be synthesised was CPP2. Only in this way would we be able to have available peptide to make the N-terminal modifications with the different thiazole derivatives. The peptide (C-terminal amide) was assembled by Fmoc/tBu SPPS methodologies. The resin (Fmoc-Rink Amide MBHA, 0.38 mmol/g loading capacity) was preconditioned for 20 min in *N*,*N*-dimethylformamide (DMF). After that, DMF was rejected, and the resin was swelled in dichloromethane (DCM) for another 15 min. The initial deprotection step (release of the free amino group of the Rink linker) was carried out using 20% piperidine in DMF (3 mL, 1 × 1 min + 1 × 20 min). After the deprotection, the resin was washed with DMF (3 mL, 3 × 1 min) and DCM (3 mL, 3 × 1 min) and a ninhydrin test was performed. Upon a positive ninhydrin test (dark blue resin beads and solution), the release of the free amine groups was confirmed and, therefore, the construction of the peptide chain could be initiated. Before coupling, amino acids were activated in solution for approximately 5 min before being transferred to the syringe to react with the resin or peptidyl-resin. This activation was accomplished by preparing a solution of Fmoc-AA-OH (5 eq.), coupling agent HBTU (5 eq.) and base DIEA (10 eq.) in DMF. Solvents were used in only sufficient volumes to ensure the proper solvation of the amino acids and the peptidyl resin beads, which is a crucial condition for efficient chain assembly. The activated amino acid solution was then transferred to the reaction vessel to react with the previously deprotected resin or peptidyl-resin for 1 h, under stirring. After this, the peptidyl-resin was washed with DMF (3 mL, 3 × 1 min) and DCM (3 mL, 3 × 1 min) to eliminate the excess of reagents, solvents and side products, and a Kaiser test was performed to confirm the efficiency of the coupling. When the Kaiser test was negative, the following deprotection step was carried out using the deprotection solution (20% piperidine in DMF (3 mL, 1 × 1 min + 1 × 20 min)). Once deprotection was completed, the resin was washed again with DMF (3 mL, 3 × 1 min) and DCM (3 mL, 3 × 1 min), and another Kaiser test was performed. When the deprotection was confirmed by a positive Kaiser test, the next Fmoc-AA-OH was coupled following the same aforementioned conditions. The peptide sequence was completed in the C→N direction by repeating the cycles of coupling of N-Fmoc amino acids and deprotection steps. When the peptide sequence was completed and after the final deprotection, the peptidyl-resin was subjected to a chemical treatment that cleaved the bond linking the peptide to the resin beads. First, a cleavage cocktail was prepared in the hood, containing 95% TFA, 2.5% H_2_O and 2.5% thioanisole (TIS) (*v/v*). Then, the dry peptidyl-resin was transferred to 15 mL Falcon tubes in 100 mg portions, and 1 mL of cleavage cocktail was added to each portion. The tubes were left under orbital stirring for 2 h at room temperature. After that, the peptide should be soluble in the solution and, thus, the contents of the tubes were filtered with a D4 funnel previously rinsed with TFA, and the resin beads were washed with TFA. The filtrate, containing the soluble peptide, was transferred to new Falcon tubes in 1 mL portions, and 14 mL of cold tert-butyl methyl ether were added to each tube. After slightly shaking the tubes, they were cooled to −22 °C for approximately 8 min and then centrifuged at 3500 rpm for 7 min at −5 °C. The ether was carefully rejected and “fresh” ether was added again. The addition of ether and centrifugation was repeated three more times and, finally, the tubes were left in a vacuum desiccator until the crude peptide was dry. Dry peptide pellets were then solubilised in 10% aqueous acetic acid and analysed by HPLC and LC-MS.

#### 4.1.3. Synthesis of the NTZ-C2-CPP2 and MTZ-C2-CPP2 Conjugates

To obtain these two conjugates, the last amino acids of the base sequence of CPP2 was deprotected and a Kaiser test was performed. When the deprotection was confirmed by a positive Kaiser test, a linker (C2) was coupled to this sequence. This step was carried out using succinic anhydride (10 eq.) and pyridine (2 mL) for 1 h under stirring. After this step, the resin was washed with DMF (3 mL, 3 × 1 min) and DCM (3 mL, 3 × 1 min) and divided into two halves. The 2-amino-5-nitrothiazole (NTZ) was coupled to one half and the 2-amino-5-methylthiazole (MTZ) to another using the coupling TBTU (5 eq.) and base DIEA (10 eq.) in DMF for 2 h under stirring. Upon synthesis, the conjugates were cleaved and, once the products were dried in a vacuum desiccator, analysed by HPLC and LC-MS.

#### 4.1.4. Synthesis of the BenzoTZ-C2-CPP2 Conjugate

This strategy was based on the synthesis in solution of the derivative BenzoTZ-C2 and subsequent coupling to the peptide in the syringe. To this end, a suspension of BenzoTZ (0.2500 g) and C2 (2.4 eq.) in DMF (3 mL) was prepared at room temperature under magnetic stirring. DIEA (2 eq.) was added and allowed to react under these conditions for 24 h. The reaction was monitored by TLC using a dichloromethane/acetone ratio of 4:1 as the eluent. Analyses were made for ESI-MS, the expected compound was confirmed to be obtained, and column purification was carried out. The tubes corresponding to the desired purified compound were selected and placed into a flask to be evaporated. The compound was then solubilised in water/methanol and lyophilised. Out of the syringe, in a small bottle, 2 eq. of purified BenzoTZ-C2, 2 eq. Of PyBOP and 4 eq. of DIEA were placed together. Then, this solution was placed in the syringe and left overnight under shaking. After 24 h, the resin was cleaved and analysed by LC-MS to confirm the formation of the conjugate.

#### 4.1.5. Synthesis of the BTZCA-CPP2 and BTZ5CA-CPP2 Conjugates

To obtain these conjugates, the last amino acid of the base sequence of CPP2 was deprotected and a Kaiser test was performed. When the deprotection was confirmed by a positive Kaiser test, the resin was washed with DMF (3 mL, 3 × 1 min) and DCM (3 mL, 3 × 1 min) and divided into two halves. Then the 5-bromothiazole-2-carboxylic acid (BTZCA, 5 eq.) and the 2-bromothiazole-5-carboxylic acid (BTZ5CA, 5 eq.) were coupled using the coupling agent TBTU (5 eq.) and base DIEA (10 eq.) in DMF for 2 h under stirring. A Kaiser test was then performed to confirm the efficacy of the coupling. Upon synthesis, the conjugates were cleaved to separate the peptide from the resin and to remove the sidechain protecting groups. Once the products were dried in a vacuum desiccator, they were analysed by HPLC and LC-MS, and it was found to correspond to the target conjugates.

#### 4.1.6. General Analysis Procedure

The CPP2 and CPP2–thiazole conjugates’ purity were determined by high-performance liquid chromatography with diode array detection (HPLC-DAD) on Merck–Hitachi LaChrom Elite equipment with a quaternary pump, automatic and thermostated by a Peltier effect injector and a diode detector. A reverse-phase Purospher star RP C-18 (octadecylsilane) column (125 × 4.0 mm), with a particle diameter of 5 μm, was used. The elution was performed with a variable gradient of acetonitrile (ACN) in water containing 0.05% trifluoracetic acid (TFA), at a 1 mL/min flow and detection at a variable wavelength (220 or 270 nm). Synthetic crudes were purified by reverse-phase medium-pressure liquid chromatography (RP-MPLC), using a C18 Vydac^®^ 218TP stationary phase, by Grace Vydac. MS analysis was performed on an LTQ Orbitrap™ XL hybrid mass spectrometer (Thermo Fischer Scientific, Bremen, Germany) controlled by LTQ Tune Plus 2.5.5 (Thermo Fischer Scientific, Bremen, Germany) and Xcalibur 2.1.0 (Thermo Fischer Scientific, Bremen, Germany). The electrospray ionisation source settings were as follows: source voltage, 3.1 kV; capillary temperature, 275 °C with a sheath gas flow rate at 40 and auxiliary gas flow rate at 10 (arbitrary unit as provided by the software settings). The capillary voltage was 36 V, and the tube lens voltage was 110 V. The direct electrospray ionisation-ion trap mass spectrometry (ESI-IT MS) analysis was performed on a Finnigan Surveyor LCQ DECA XP MAX mass spectrometer from ThermoElectron Corporation (Waltham, MA, USA), and the LC-MS analysis was performed on the same equipment coupled to a Finnigan Surveyor HPLC equipped with a DAD Plus Detector, an Autosampler Plus and an LC Pump Plus. MALDI-TOF/TOF matrix-assisted desorption/ionisation mass spectrometry was performed using a Bruker Daltonics UltrafleXtreme (Bruker, MA, USA). MS data handling software (Xcalibur QualBrowser software, Thermo Fischer Scientific, Bremen, Germany) was used to obtain the confirmation of the synthetic peptides by their exact *m*/*z* value.

### 4.2. Cell Culture

#### 4.2.1. Materials

McCoy’s 5A Modified Medium, Dulbecco’s modified Eagle medium (DMEM), dimethyl-sulfoxide (DMSO), foetal bovine serum (FBS) and a penicillin–streptomycin solution were purchased from Millipore Sigma (Merck KGaA, Darmstadt, Germany). Other cell culture reagents were purchased from Gibco (Thermo Fisher Scientific, Inc., Waltham, MA, USA). 5-FU (cat. no. F6627), CLZ (cat. no. C6019), thiazolyl blue tetrazolium bromide (MTT, cat. no. M5655) and sulforhodamine B (SRB, cat. no. S1402) were obtained from Sigma–Aldrich (Merck KGaA, Darmstadt, Germany). PTX (cat. no. 1097) was obtained from Tocris Bioscience (Bristol, UK).

#### 4.2.2. Cell Line and Cell Culture

Human colorectal cancer HT-29 and prostate cancer PC-3 cell lines were obtained from the American Type Culture Collection (ATCC; Manassas, VA, USA) and maintained according to ATCC’s recommendations at 37 °C and 5% CO_2_ in appropriate medium supplemented with 10% foetal bovine serum, 100 U/mL penicillin G and 100 µg/mL streptomycin. Cells were maintained in the logarithmic growth phase at all times. The media were changed every 2 days and trypsinised with 0.25% trypsin–EDTA. A total of 100 µL of HT-29 cells (7500 cells/well) or PC-3 cells (5000 cells/well) were seeded in 96-well plates and allowed to adhere overnight before drug exposure. After 24 h, the cell culture media were replaced with 100 µL of drug-containing media. Cells were exposed to drugs/peptides for 48 h, followed by MTT and SRB assays to evaluate single and combination drug treatments in the cell viability and protein synthesis rate of these cells.

#### 4.2.3. Cell Culture Treatment

The IV value was first determined for each drug alone in HT-29 and PC-3 cells. Drug/peptide concentrations ranged from 0.1 to 50 µM for the single-drug treatment. Combination studies were performed by combining 5-FU or PTX (Drug A), according to each cell line, with CLZ or each CPP2–thiazole conjugate (Drug B). CLZ was also combined with each CPP2–thiazole conjugate. Drug A was 5-FU for HT-29 cells and PTX for PC-3 cells. Only peptides that present the most promising pharmacological profile (IV < 20 µM) were tested in simultaneous combination with 5-FU, PTX or CLZ. Both Drug A and Drug B concentrations were variable, and the combined effects of equipotent concentrations (fixed ratio) of the IV values for each drug were evaluated.

#### 4.2.4. Cytotoxicity Assays

To determine the effects of 5-FU/PTX, CLZ and each CPP2–thiazole derivate on the viability of HT-29 and PC-3 cells, MTT and SRB assays were used. For the MTT protocol, after drug treatment, the cell medium was removed and 100 µL/well of MTT solution (0.5 mg/mL in PBS) was added. Cells were incubated for 3 h, protected from light. After this period, the MTT solution was removed, and DMSO (100 µL/well) was added to solubilise the formazan crystals. Absorbance was measured at 570 nm in an automated microplate reader (Tecan Infinite M200, Tecan Group Ltd., Männedorf, Switzerland). For the SRB assay, after treatments, the cultured cells were fixed with ice-cold 10% trichloroacetic acid for 30 min and stained with 0.4% SRB for 1 h at room temperature. Excess dye was removed by rinsing several times with tap water. Protein-bound dye was dissolved with 200 µL 10 mM Tris base solution for the determination of absorbance with a microplate reader with a filter wavelength of 540 nm (Tecan Infinite M200, Tecan Group Ltd., Männedorf, Switzerland). The IV of the therapeutic drug was determined as each drug concentration showing 50% cell growth inhibition compared with the control. All conditions were performed three times independently in triplicate.

#### 4.2.5. Cell Morphology Visualisation and Cell Count Analysis

After each treatment, cell morphology was assessed on a Leica DMI 6000B microscope equipped with a Leica DFC350 FX camera and then analysed with the Leica LAS X imaging software (v3.7.4, Wetzlar, Germany).

#### 4.2.6. Data Analysis

GraphPad Prism 8 (GraphPad Software Inc., San Diego, CA, USA) was used to produce concentration–response curves by nonlinear regression analysis. The viability of cells treated with each drug was normalised to the viability of control cells and cell viability fractions were plotted vs. drug concentrations in the logarithmic scale.

#### 4.2.7. Analysis of Drug Interactions

To quantify drug interaction in each combination, we first estimated the combination index (CI) by the unified theory, introduced by Chou and Talalay [20], using the CompuSyn software (ComboSyn, Inc., New York, NY, USA). We used the mutually exclusive model, based on the assumption that drugs act through entirely different mechanisms [21]. The two drugs were combined in a fixed ratio of doses that corresponded to 0.25, 0.5, 1, 2 and 4 times that of the individual IV values. CI was plotted on the *y*-axis as a function of the effect level (Fa) on the *x*-axis to assess drug synergism between drug combinations. The CI is a quantitative representation of pharmacological interactions. CI < 1 indicates synergism, CI = 1 indicates additive interaction and CI > 1 indicates antagonism.

#### 4.2.8. Statistical Analysis

The results are presented as mean ± SEM for n experiments performed. All data were assayed in three independent experiences, in triplicate. Statistical comparisons between control and treatment groups, at the same time point, were performed with Student’s *t*-test and one-way ANOVA test. Statistical significance was accepted at *p* values < 0.05.

## Figures and Tables

**Table 1:** Cytotoxicity of 5-FU, PTX, clotrimazole and CPP2–thiazole conjugates in HT-29 and PC-3 cells. IV values are given as the mean and were obtained by nonlinear regression using GraphPad software.

Drug	HT-29 IV (µM)	PC-3 IV (µM)
5-FU	2.58	ND
PTX	ND	0.01
Clotrimazole	21.64	14.08
CPP2	>50	>50
BenzoTZ-C2–CPP2	0.21 ^⁑^	0.84 ^⁑^
NTZ-C2–CPP2	>50	0.49 ^⁑^
MTZ-C2–CPP2	>50	0.06 ^⁑^
BTZCA–CPP2	0.05 ^⁑^	0.003 ^⁑^
BTZ5CA–CPP2	0.01 ^⁑^	>50

ND: not determined; ^⁑^ selected for combination with CLZ or antineoplastic drug.

## Data Availability

Not applicable.
